# Microstructural characterization of myocardial infarction with optical coherence tractography and two‐photon microscopy

**DOI:** 10.14814/phy2.12894

**Published:** 2016-09-20

**Authors:** Craig J. Goergen, Howard H. Chen, Sava Sakadžić, Vivek J. Srinivasan, David E. Sosnovik

**Affiliations:** ^1^ Weldon School of Biomedical EngineeringPurdue UniversityWest LafayetteIndiana; ^2^ Athinoula A. Martinos Center for Biomedical ImagingMassachusetts General HospitalHarvard Medical SchoolCharlestownMassachusetts; ^3^ Cardiovascular Research CenterDepartment of MedicineMassachusetts General HospitalHarvard Medical SchoolCharlestownMassachusetts; ^4^ Department of Biomedical EngineeringUniversity of California DavisDavisCalifornia

**Keywords:** Fiber architecture, myocardial infarction, Myocardium, optical coherence tomography, tractography, two‐photon microscopy

## Abstract

Myocardial infarction leads to complex changes in the fiber architecture of the heart. Here, we present a novel optical approach to characterize these changes in intact hearts in three dimensions. Optical coherence tomography (OCT) was used to derive a depth‐resolved field of orientation on which tractography was performed. Tractography of healthy myocardium revealed a smooth linear transition in fiber inclination or helix angle from the epicardium to endocardium. Conversely, in infarcted hearts, no coherent microstructure could be identified in the infarct with OCT. Additional characterization of the infarct was performed by the measurement of light attenuation and with two‐photon microscopy. Myofibers were imaged using autofluorescence and collagen fibers using second harmonic generation. This revealed the presence of two distinct microstructural patterns in areas of the infarct with high light attenuation. In the presence of residual myofibers, the surrounding collagen fibers were aligned in a coherent manner parallel to the myofibers. In the absence of residual myofibers, the collagen fibers were randomly oriented and lacked any microstructural coherence. The presence of residual myofibers thus exerts a profound effect on the microstructural properties of the infarct scar and consequently the risk of aneurysm formation and arrhythmias. Catheter‐based approaches to segment and image myocardial microstructure in humans are feasible and could play a valuable role in guiding the development of strategies to improve infarct healing.

## Introduction

The orientation of cardiac myofibers plays a key role in both electrical conductance and contraction of the left ventricle. Histological studies have shown that myofibers form a series of crossing spiral structures in healthy myocardium (Streeter et al. 1969). Specifically, fibers in the subepicardium form a left‐handed helix, while fibers in the subendocardium form a right‐handed helix (Streeter and Hanna 1973). Histological studies, while of major value, cannot assess tissue microstructure in intact organs or in vivo. Diffusion weighted magnetic resonance imaging (MRI), in contrast, has been extensively used to characterize the microstructure of normal and infarcted hearts ex vivo (Scollan et al. 1998; Chen et al. 2003; Wu et al. 2007; Strijkers et al. 2009) and in vivo (Reese et al. 1995; Tseng et al. 1999; Wu et al. 2006). Moreover, techniques to perform diffusion MRI tractography of the heart have been developed. These have been used to characterize myofiber architecture in infarcted hearts ex vivo (Sosnovik et al. 2009) and more recently in vivo as well (Sosnovik et al. 2014).

DTI tractography of the heart in vivo has several advantages (Sosnovik et al. 2014). It allows the microstructure of the myocardium to be examined in three dimensions, serially over extended periods of time, and under normal loading conditions (Sosnovik et al. 2014). The limitations of DTI‐tractography, however, include its spatial resolution (0.1–1 mm) and inability to resolve molecular signatures in the tissue imaged. Optical imaging techniques combine several of the advantages of histology and DTI‐tractography. They allow intact tissues to be imaged with micron‐scale resolution in 3D. In addition, properties such as absorption, birefringence, scattering, and fluorescence can be used to characterize the cellular and molecular composition of the tissue.

Optical coherence tomography (OCT) has been used to create tracts and quantify cardiac fiber orientation in normal mice (Goergen et al. 2012), rabbits (Fleming et al. 2008), and with a 3D approach in pigs (Gan and Fleming 2013). Others have used polarization‐sensitive OCT to determine fiber orientation in both neural (Wang et al. 2011) and cardiac (Fan and Yao 2013; Wang and Yao 2013) tissue. Two‐photon microscopy (TPM) has been used to image collagen fibers via second harmonic imaging (Tsai et al. 2010) and fiber orientation via autofluorescence.

The purpose of this study was to use a combined OCT/TPM approach to characterize fiber architecture in myocardial infarction. The microstructural relationship between collagen and cardiac myofibers is not fully understood, but is likely to be especially important after ischemic injury. Using diffusion MRI‐tractography, we have previously demonstrated the presence of a large number of residual myofibers in the infarct zone of rats with myocardial infarction. We aimed here to more fully evaluate microstructure in infarcted myocardium and characterize the relationship between residual myofibers and the collagen fiber network in the infarct zone.

## Methods

### Animal model

All procedures were performed with local Institutional Animal Care and Use Committee approval. The C57BL/6 mice used in this study were between 12 and 14 weeks old and anesthetized with 1–3% isoflurane in 2 L/min O_2_ during the surgical procedures. We used a murine left coronary artery permanent ligation model to induce myocardial infarction (Sosnovik et al. 2009). Animals were euthanized 25–26 days after surgery, their hearts removed, and blood was rinsed away by submerging the tissue in DPBS. A second group of age‐matched C57BL/6 mice that did not undergo surgery were used as a control. OCT was performed on tissue from six mice in both the infarcted and control groups, while two‐photon imaging was performed on tissue from eight animals in both groups. Cardiac tissue was submerged in an optimal cutting temperature compound‐embedding medium, frozen, and then thawed at room temperature. We empirically found that this procedure improves OCT image contrast. The hearts were then optically cleared by placing them in a 50% glycerol solution for 24 h (Sigma Chemical, St. Louis, Missouri). A glass coverslip was placed on the anterolateral wall of the left ventricle and used to lightly compress the tissue, creating a flat perpendicular surface for optical imaging. Finally, histology was performed by embedding the tissue in paraffin, sectioning 5‐μm‐thick slices parallel to the epicardial surface at three different levels, and then staining with hematoxylin and eosin (H&E).

### OCT imaging system

Optical coherence tomography was performed with a 1310 nm spectral/Fourier domain system custom‐built on a Nikon microscope platform to acquire image volumes (Michael et al. 1995). Two unpolarized superluminescent diodes were combined with a 50/50 fiber coupler to create a light source with a bandwidth of 170 nm. The axial scanning speed was 47,000 axial scans per second achieved by a 1024 pixel InGaAs line scan camera (Goodrich‐Sensors Unlimited). The axial resolution was approximately 4.7 μm in air. OCT was performed with a 10× objective that enabled a transverse resolution of 3.6 μm FWHM. Volumes consisting of 768 frames with 1024 axial scans per frame were acquired over 1.5 mm × 2 mm regions.

### OCT image analysis

Optical coherence tomography data were collected from six infarcted and six control hearts through the entire thickness of the left ventricle. For the OCT data, subepicardium was defined as the outer third of the myocardium, Attenuation of the OCT signal with depth was quantified in three regions: (1) infarct core; (2) infarct edge; and (3) normal control myocardium. We manually defined a region of interest over the brightest section at focus depths every 105 μm. The slope of signal v focus depth, or attenuation (dB/μm), was calculated for all three regions.

2D tractography of the OCT data was performed as previously described (Goergen et al. 2012). Briefly, a 2D discrete Fourier transform (DFT) was calculated after multiplication by a 0.36 mm symmetric Hanning window that was scanned across the original image slice in step sizes of 0.09 mm. Local orientation distribution functions (ODFs) were constructed based on the sum of the high‐pass filtered 2D DFT slice magnitude squared at each angle.

(1) $$\text{ODF}\left(\theta \right)=\int_{-\infty }^{\infty }{\left|F_{w}\left(f_{x},f_{y}\right)\right|}^{2}H\left(f_{x},f_{y}\right)df$$

where *f*_*x*_ is −*f* sin (*θ*), *f*_*y*_ is −*f* cos (*θ*), *F*_*w*_ is the windowed Fourier transform, and ***H*** is a high‐pass filter. The maximum of the ODF gives the local fiber orientation, and multiple maxima indicate multiple orientations within the windowed region. The length of each streamline was scaled based on the ratio of the peak of the ODF to the variance of the ODF 90° away from the peak. The ODF field was then used to construct tracts or streamlines that originated from each point and then propagated in both directions of the ODF maximum. The direction was updated by multiplying the ODF by a circularly Gaussian window centered on the orientation from the previous iteration. The fiber inclination angle at each depth was calculated by averaging the direction of all streamlines estimated from the starting and ending points. Angular data *θ*_*n*_ were characterized using the circular mean ($\overset{¯}{\theta }$) and variance (Var), defined using circular statistics and accounting for modulo 180° equivalence.

(2) $$\overset{¯}{\theta }=\frac{1}{2}\text{Arg}\left(\frac{1}{N}\sum_{n=1}^{N}e^{i2\theta_{n}}\right)$$

(3) $$\text{Var}=1-\sqrt{{\left[\frac{1}{N}\sum_{n=1}^{N}\text{cos}\left(2\theta_{n}\right)\right]}^{2}+{\left[\frac{1}{N}\sum_{n=1}^{N}\text{sin}\left(2\theta_{n}\right)\right]}^{2}}$$

By definition, all angles fall between −90° and 90°.

### Two‐photon imaging system

Two‐photon imaging (Goergen et al. 2012) was performed with a commercial laser‐scanning microscope (Ultima, Prairie Technologies, Inc., Middleton, Wisconsin). A pair of conventional nonresonant galvanometer‐based scanners moved a single optical beam in the horizontal plane. A motorized stage controlled the fine positioning of the microscope objective along the optical axis. Emission from the sample was reflected by a high‐pass dichroic mirror and detected by a four‐channel detector. A dichroic mirror split the emission light into two arms. Each arm contained a pair of photomultiplier tubes (PMTs) and a filter cube. The emission filter transmission windows were 460 ± 25 nm for second harmonic generation (SHG) and 525 ± 25 nm for autofluorescence. Microscopic imaging was conducted with an Olympus XLUMPLFL water‐immersion 20× objective that had a 2 mm working distance [numerical aperture (NA) = 0.95]. We generated the 920 nm excitation beam with a femtosecond laser (Mai‐Tai, Spectra‐Physics, Irvine, California). An electro‐optic modulator controlled the optical power on the sample (Conoptics Inc., Danbury, Connecticut). For the two‐photon data, we defined the subepicardium as the region of myocardium extending 200 μm deep from the epicardial (outer) surface of the heart, which corresponded to the typical penetration depth of the system. Individual images were combined to create images of composite, SHG, and autofluorescence signal using a stitching plugin and linear blending within Fiji (Zipfel et al. 2003).

### Statistics

Data are presented as mean ± standard deviation. Statistical significance between groups was determined with a Student's *t*‐test when comparing infarcted and control tissue. An analysis of variance (ANOVA) with a Tukey–Kramer honestly significant difference post hoc test was used when comparing multiple groups from the infarct core, infarct edge, and control tissue (JMP 12.0.1, SAS Institute Inc., Chicago, IL).

## Results

### Depth dependence of myofiber direction

**Figure 1:**
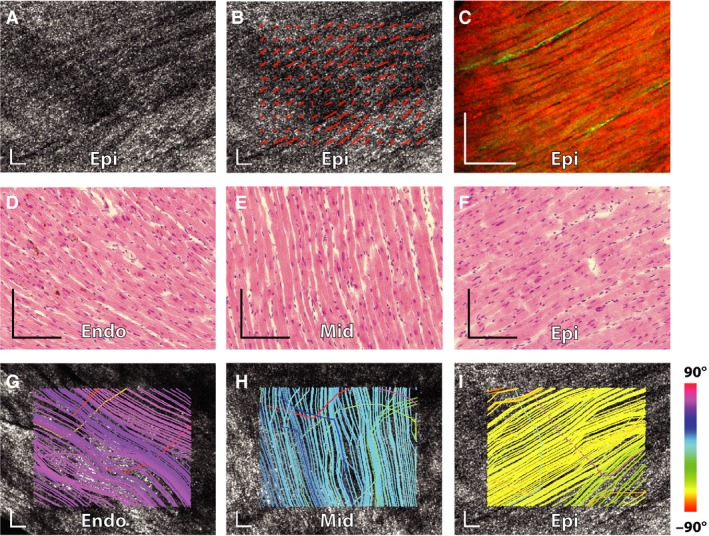
Myofiber direction changes with depth in a control murine heart. (A) Representative *en face* OCT image shows fibers forming a left‐handed helix in the subepicardium of the heart. This direction was confirmed with ODF analysis (B) and two‐photon microscopy (C). Histological H&E images from the endocardium (D), midwall (E), and epicardium (F) reveal changes in fiber angle with depth that are also seen in the tractography streamline analysis from the same levels (G–I). Scalebars represent 100 μm.

**Figure 2:**
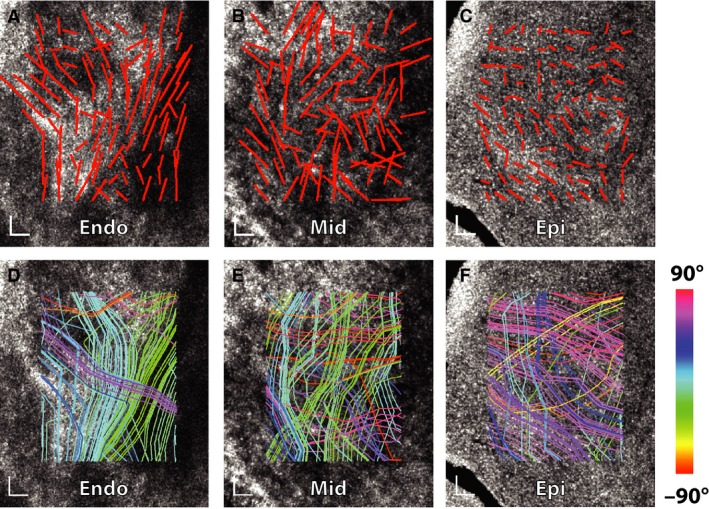
OCT images of a mature infarct scar reveal severe disorder. Streamline analysis is shown in the endocardium (A), midwall (B), and epicardium (C). Individual streamlines are combined to form tracts that suggest severe disarray (D–F). Scalebars represent 100 μm.

The image features in en face OCT images were locally elongated along the fiber axis, as shown without (Fig. 1A) and with ODF peaks (Fig. 1B). We confirmed this local morphology by imaging myocytes at a similar depth using two‐photon excited autofluorescence (red) and collagen using second‐harmonic generation (green) as seen in Figure 1C from the same specimen. Both the H&E stained histological sections (Fig. 1D–F) and OCT tractography streamlines (Fig. 1G–I) of the endocardium, midwall, and epicardium showed clear changes in myofiber direction from +60° to −60°. In contrast, OCT tractography of the infarcted hearts revealed marked disorder in the infarct zone with incoherent tracts running in multiple directions and showing no clear change with depth (Fig. 2). This feature allowed normal myocardium to be distinguished from infarcted myocardium with a high degree of accuracy.

### Quantification of tract orientation

**Figure 3:**
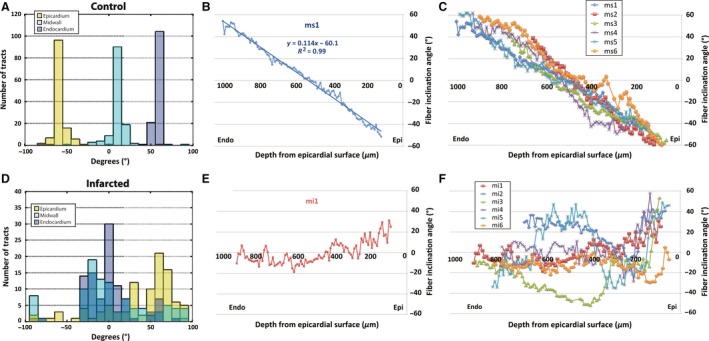
Quantification of cardiac tract orientation before and after infarction. A histogram from a representative control heart reveals sharp peaks from tracts acquired in the epicardium, midwall, and endocardium (A), with a smooth linear transition in angle with tissue depth (B). This result was consistent for all six control hearts (C). A representative infarcted heart shows no substantial peaks in a similar histogram (D) and altered fiber angles with respect to tissue depth (E). All six infarcted hearts showed a similar lack of coherence and transmural fiber organization (F).

Myofiber orientation in control hearts was highly coherent at a given transmural depth. A linear gradient in fiber orientation was seen from the subepicardium to the subendocardium. This is shown in (Fig. 3A), where the mean epicardial, midwall, and endocardial directions in the heart imaged were −56.3°, 9.3°, and 55.7° respectively, with small circular variances (epicardial: 0.11, midwall: 0.20, and endocardial 0.13). A similar pattern was seen in all six control animals (Fig. 3B–C). Infarcted tissue, on the other hand, displayed a marked degree of structural incoherence with OCT. For one representative infarcted heart (Fig. 3D), the mean epicardium, midwall, and endocardial directions for all fibers (myofiber and collagen) were 31.2°, 1.1°, and 0.02° respectively, with large circular variances (epicardial: 0.61, midwall: 0.79, and endocardial: 0.48). No consistent changes in mean fiber direction with depth were observed in any of the six infarcted hearts (Fig. 3E–F).

**Figure 4:**
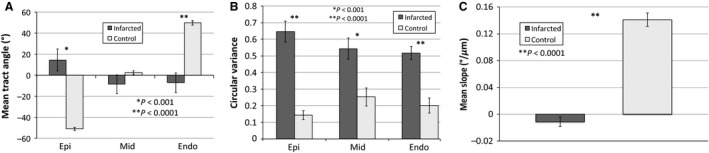
Analysis of cardiac fiber orientation before and after infarction. The mean tract angle was significantly different between the control and infarcted hearts at representative epicardial and endocardial levels (A) and the circular variance was significantly increased in infarcted hearts for all three depths (B). The mean slope of fiber angle versus depth from the epicardial surface for the six infarcted hearts was significantly lower than the six control hearts (C). *P < 0.001 and **P < 0.0001.

The mean tract angle of all fibers for both groups at three different levels is shown in Figure 4A. The control group showed a clear and distinct transition from ‐51.2 ± 1.4° in the epicardium to 2.3 ± 1.7° in the midwall and 49.7 ± 2.2° in the endocardium. The corresponding values in the infarcted hearts were epicardium 14.4 ± 10.4°, midwall −8.6 ± 8.8°, endocardium −7.2 ± 9.4°. Infarcted myocardium had tracts that were highly random with high circular variance (Fig. 4B). Circular variance in the control hearts was 0.14 ± 0.03 in the epicardium, 0.25 ± 0.05 in the midwall, and 0.20 ± 0.05 in the endocardium, which was significantly lower than the infarcted hearts (epicardium 0.65 ± 0.06, midwall 0.54 ± 0.06, endocardium 0.52 ± 0.04). The mean slope of fiber inclination angle versus transmural depth is shown for each animal (Figs 3C and 4F). The control animals had a significantly higher slope of 0.141 ± 0.01°/μm compared to −0.011 ± 0.01°/μm in the infarcted group.

### OCT signal attenuation increases after infarction

**Figure 5:**
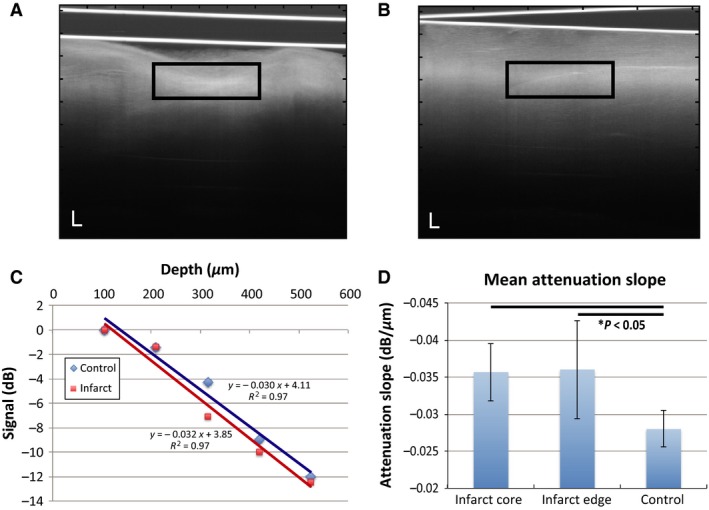
OCT signal attenuates more quickly in infarcted myocardium. Representative OCT images illustrating focal planes as a bright region within myocardial tissue for both infarcted (A) and control (B) tissue. The black boxes represent the regions roughly 200 μm below the surface where the signal was quantified. (C) Typical signal attenuation with depth within both the infarct core and normal control myocardium illustrate the difference in attenuation for the healed infarct compared to healthy control regions. (D) The mean slope ± SD is significantly higher in infarcted regions (*P < 0.05). Scalebars represent 100 μm.

The OCT signal in infarcted regions attenuated more quickly with depth when compared to normal regions (Fig. 5). The mean signal attenuation slope was significantly higher (*P* < 0.05) in infarcted tissue (core: −0.036 ± 0.004 dB/μm; edge: −0.036 ± 0.007 dB/μm) than control tissue (−0.028 ± 0.002 dB/μm). No significant difference was observed between the edge and core of the infarction. These attenuation measurements are consistent with a high degree of collagen increasing light attenuation in the infarct, and is an expected finding 4 weeks after coronary ligation.

### TPM of infarcted myocardium and residual myofibers

**Figure 6:**
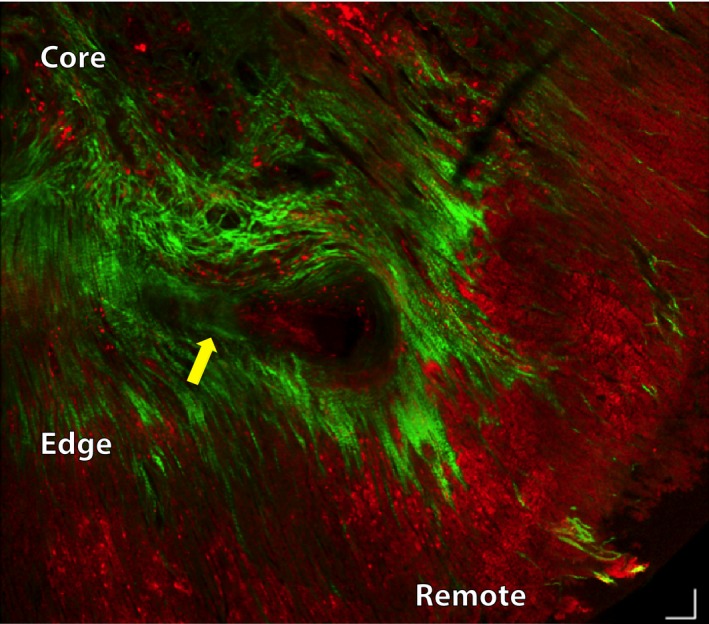
Two‐photon microscopy of the border zone of an infarct and adjacent noninfarcted myocardium. A tiled overlaid image of twelve individual locations is shown with the infarct core, edge, and remote regions labeled. The suture placed to ligate the left coronary artery can be seen in the middle of the image (yellow arrow). Normal myocardium consists predominantly of the autofluorescence signal (red) from the myocytes. The border zone of the infarct shows a substantial increase in collagen content (green) but large numbers of myofibers remain. The infarct zone has a large number of collagen fibers and fewer residual myocytes. Scalebars represent 50 μm.

Two‐photon microscopy was used to further characterize the infarct and border zones. The need for this was underscored by the high attenuation in OCT images of the infarct, and the likely presence of high amounts of collagen. The second harmonic signal produced by collagen in the TPM images is shown in green and the autofluorescence signal from myocytes in red (Fig. 6). Infarcted myocardium was characterized by a marked increase in collagen content (Fig. 6).

**Figure 7:**
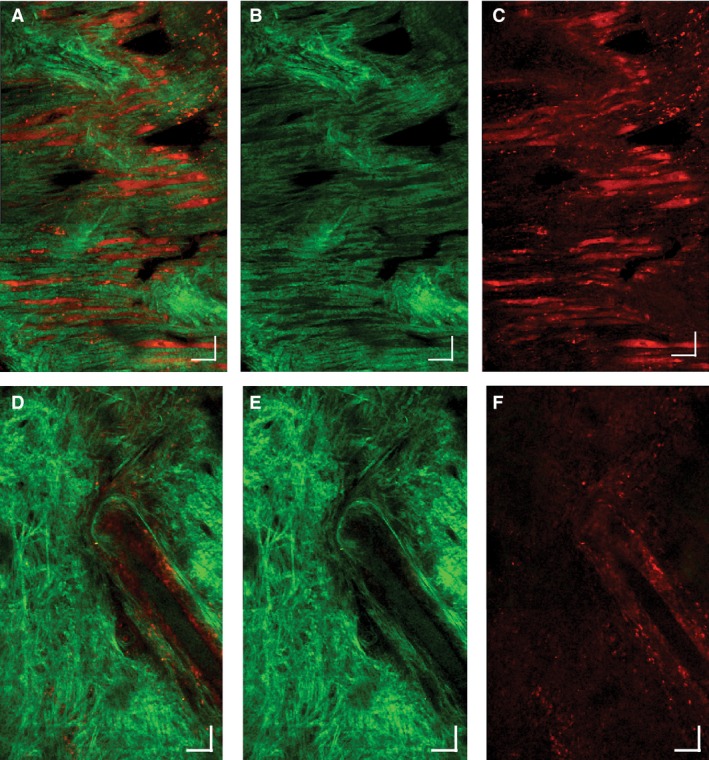
Two‐photon microscopy of regions with residual myofibers and disorganized collagen. Collagen fibers (green) in some regions were arranged in a coherent pattern parallel to myofibers (red). These residual myocytes can be clearly seen in A (merged image), B (second‐harmonic collagen signal), and C (autofluorescence myocyte) panels. A second region in the center of the infarct formed poorly organized, incoherent collagen networks that lacked any clear organization (D–F). The orientation of the collagen fibers appears random and the suture used to occlude the left coronary artery is visible in all three of the lower panels. Scalebars represent 50 μm.

Two distinct patterns of collagen architecture were seen in the infarct zone. In regions where residual myofibers were present, the collagen fibers were aligned parallel to the residual myofibers in a fairly coherent fashion (Fig. 7A–C). However, in regions of the infarct with no residual myofibers, the collagen fibers were oriented in a completely incoherent and random fashion (Fig. 7D–F). These two patterns of collagen architecture were seen in all of the infarcted hearts.

## Discussion

The importance of the microstructure of the heart is becoming increasingly recognized in cardiovascular medicine (Preibisch et al. 2009). Traditionally, the architecture of the heart has been evaluated with histology. This approach, while of major value, is destructive and vulnerable to tissue shearing, tearing and distortion. The ability to resolve 3D architecture from 2D stacks of histological sections is also challenging. Diffusion weighted MRI provides numerous advantages but lacks microscopic resolution and the ability to characterize tissues at the molecular level. Here we present a hybrid optical approach consisting of OCT and TPM to overcome these limitations.

The presence of residual myofibers in infarcted myocardium has been well documented histologically. Diffusion MRI tractography of infarcted hearts has also demonstrated that residual tracts are present in the infarct zone and correlate histologically with the presence of residual myofibers (Scollan et al. 1998; Chen et al. 2003; Wu et al. 2007; Sosnovik et al. 2009, 2014; Strijkers et al. 2009). Here we show that residual myofibers are frequently present in the infarct zone and, moreover, that they modulate the coherence of the surrounding collagen network. In the presence of residual myofibers the collagen fibers in the infarct scar align parallel to the myofibers in a coherent pattern. However, in the absence of residual myofibers, the collagen fibers in the infarct zone are randomly aligned and show no architectural coherence.

Myocardial infarction results in the loss of functional myocardium and initiates an adverse remodeling process in the heart. During remodeling the infarct thins and expands, which imposes abnormal stresses on the border zone of the infarct. Cardiomyocytes in the border zone undergo apoptosis in response to these abnormal stresses, which causes the infarct to expand further and the left ventricle to dilate. The presence of coherently organized residual cardiomyocytes in the infarct may have important implication for its tensile strength. Coherent microstructure in the infarct may improve its ability to avoid thinning and left ventricular dilation.

The nature of the microstructure in the infarct also has important implications for the risk of lethal arrhythmias. Dispersion in electrical repolarization can make the myocardium vulnerable to electrical re‐entry and ventricular tachycardia (Baker et al. 2000). Unlike with mechanical remodeling, the presence of residual myofibers in the infarct may result in adverse electrical remodeling. The properties of the action potential in the residual cardiomyocytes frequently differ from normal myocardium and can form the nidus of an arrhythmic circuit. The detection of foci of residual myocytes in the infarct could help refine the risk for sudden cardiac death and guide treatment strategies, including electrical ablation.

Optical coherence tomography allows normal myocardium to be distinguished from injured myocardium based on the presence of tract disarray. This finding, however, cannot be interpreted in isolation. If the attenuation of light in the region of tract incoherence is normal, this suggests the presence of a dense but highly disordered network of residual myofibers. This scenario might be encountered for instance in hypertrophic cardiomyopathy. The presence of increased attenuation, however, suggests possible changes in tissue composition. These changes may include the loss of blood or an increase in immune cells within the core. However, the most likely explanation for the increased attenuation is that in infarcted hearts, large portions of the myocardium have been replaced with a dense collagen network (Sosnovik et al. 2009, 2014), which can be ordered or disordered. This distinction cannot be made with OCT alone and the addition of TPM is required. The autofluorescence of myofibers and the second harmonic signal produced by collagen allows detailed characterization of both compartments to be performed with TPM (Leitgeb et al. 2000; Xu et al. 2004).

No areas of normal attenuation in the infarcts were seen in this study. Rather, all areas of tract disarray by OCT showed increased attenuation. TPM microscopy of these regions revealed two distinct patterns. Coherent areas of collagen fibers in the infarct zone were seen only in the presence of residual fibers. Portions of the infarct containing no residual myofibers showed highly irregular collagen networks. OCT was thus able to accurately distinguish coherence versus disarray in myofiber networks, but not in collagen networks. Ordered and disordered collagen networks produced a similar signature on OCT and could only be distinguished with the addition of TPM.

The limitation of optical techniques in the heart lies in their potential for translation. Both OCT and TPM require catheter based approaches to image cardiovascular structures. OCT of the coronary arteries (Tsai et al. 2010), however, is routinely performed, meaning the design of catheter‐based systems for myocardial imaging should be feasible (Leitgeb et al. 2000; Xu et al. 2004). Clinical application will preclude the use of optical clearing techniques. However, the described OCT approach can visualize fiber structure approximately 0.5 mm below the surface without optical clearing (data not shown). This would be very adequate in thin‐walled cardiovascular structures such as the left atrium, aorta and right ventricle. The human left ventricle is approximately 10 mm thick and optical approaches, regardless of the presence or absence of clearing, will be limited to surface and subsurface imaging. This, however, can still provide extremely useful information as demonstrated by electro‐anatomical voltage mapping of the left ventricle, a routinely performed procedure.

Remodeling of the left ventricle in the human and mouse heart show many similarities but also some important differences. The time course of infarct healing in humans and larger animals is far longer than it is in mice. This may account for differences in microstructure at the 4–6 week time point between our study and prior histological studies, which were conducted in humans and large animal models (Whittaker et al. 1989; Wickline et al. 1992). Optical techniques are particularly suited to mice and have the potential to resolve myocardial microstructure with excellent contrast and spatial resolution. However, full characterization of the myocardium also requires its viscoelastic properties to be resolved, which can be done with ultrasound and MR elastography but not with current optical techniques in vivo. Further study will be needed to relate changes in microstructure to changes in the viscoelastic properties of the heart. Likewise, given the differences in physiology and wound healing between the mouse and human hearts, further study will be needed to determine whether the microstructural changes in mature infarct scars are similar in mice and humans.

Tractography in this study was performed in 2D. Future work will be needed to quantify tract geometry in 3D (Jang et al. 2002). This has been done previously with OCT using a gradient based algorithm to quantify orientation and a particle filtering technique to track myofibers along their length (Singh‐Moon et al. 2015). Techniques to resolve 3D tracts from TPM datasets will also need to be developed. In addition, the use of segmented OCT maps and targeted fluorescent imaging agents has the potential to improve the value of OCT and TPM even further.

## Conclusion

Optical techniques including OCT are frequently used to image atherosclerotic plaques. Here, we show that a similar approach can be used to characterize myocardial microstructure in infarcted hearts. Segmented TPM images show that collagen fibers in the infarct display structural coherence only when adjacent to residual myofibers. Future work will be required to develop improved 3D tractographic techniques based on segmented 3D tissue maps. While the current study focuses on the imaging of intact tissues ex vivo, the translation of the approach with catheter‐based techniques is highly feasible.

## Competing Interests

None declared.
